# An Emerging Dimension in Psychosomatic Research: The Nocebo Phenomenon in the Management of Chronic Pain

**DOI:** 10.1155/2013/574526

**Published:** 2013-02-12

**Authors:** A. Ciaramella, M. Paroli, P. Poli

**Affiliations:** Pain Therapy Unit, Department of Oncology, Azienda Ospedaliero-Universitaria Pisana, Via Roma 67, Pisa 56127, Italy

## Abstract

*Introduction*. The nocebo effect consists in delivering verbal suggestions of negative outcomes so that the subject expects clinical worsening. Several studies indicate that negative verbal suggestions may result in the amplification of pain. Amplification style is one of the most important dimensions in psychosomatic research. *Methods*. One group of pain therapy unit patients was evaluated at baseline and again after 6 months from the beginning of the pain treatment. *Results*. Only 43% of 86 chronic pain patients respond positively to the expectation of sham pain. This group shows at baseline higher pain intensity (*t* value: 2.72, *P* = 0.007) and lower cold pain threshold (*t* value: 2.18, *P* = 0.03) than the group of subjects with any response to sham pain stimulus. Somatoform dimensions influence positively the strength of nocebo response in those predisposed to it. *Conclusion*. Our study shows that the power of the nocebo phenomenon seems to be a dimension belonging to the investigation in psychosomatic. In contrast to what one might expect, the presence of the nocebo phenomenon affects positively pain relief and the outcome of pain treatment. In a clinical setting, and the meaning of nocebo response does not seem to be different from placebo response.

## 1. Introduction

The nocebo effect consists in delivering verbal suggestions of negative outcomes so that the subject expects clinical worsening [1]. Several studies indicate that negative verbal suggestions may result in the amplification of pain [2, 3] and in the alteration of somatosensory perception [4]. In order to try to disentangle the effects of positive and negative cues on pain processing, several authors used brain imaging [5–7]. Expectation of pain increase has been found to enhance the activation of the thalamus, insula, prefrontal cortex, and anterior cingulate cortex [6], but it is not known how this variation may influence pain processing in clinical pain [8]. Previous studies suggest that nocebo effects, sometimes termed “negative placebo effects,” contribute appreciably to a variety of medical symptoms [9, 10] adverse events in clinical trials and medical care [11–14], and public health “mass psychogenic illness” outbreaks [15]. While the majority of studies on placebo and nocebo effects show consistent results with patient expectations and the main psychological mechanisms, they seem to be the related factors of subconscious conditioning and conscious expectations [8].Kennedy introduced the concept of “nocebo reaction” in 1961, a few years after Beecher published his landmark paper on the placebo effect. Kennedy attributed it to a “quality inherent in the patient, not the remedy” [16, 17].

The DSM category of “psychological factors affecting medical condition” had virtually no impact on clinical practice. However, several clinically relevant psychosomatic syndromes have been described in the literature: disease phobia, persistent somatization, conversion symptoms, illness denial, demoralization, and irritable mood which are called Diagnostic Criteria for Psychosomatic Research (DCPR) and which are described in the previous versions of DSM (Diagnostic Statistical Manual for Mental Disorder), a *psychophysiologic* characteristic that affect doctor-patient relationship [18]. These syndromes, in addition to the DSM definition of hypochondriasis, can yield clinical specification in the category of “psychological factors affecting medical condition” and eliminate the need for the highly criticized DSM classification of somatoform disorders. This new classification is supported by a growing body of research evidence and is in line with psychosomatic medicine as a recognized subspecialty [19].

We investigate the relationship between perception and management of clinical pain and the nocebo response induced by a sham pain stimulus. Relationship between some of DCPR dimensions and nocebo phenomenon and their impact on the management of chronic pain has also been investigated. 

## 2. Materials and Methods

### 2.1. Sample

Participants were recruited consecutively from the clinic of the Pain Therapy Unit of Santa Chiara Hospital in Pisa before starting pain treatment. Everyone signed the informed consent at baseline and an ethical approval has been obtained from our Ethical Committee. All patients signed the informed consent before enrolling. 

A telephone call was performed after 6 months from the baseline to investigate whether patients were still in treatment or not. At this contact, patients rate the intensity of pain (using NRS) that they felt at that time.

### 2.2. Psychophysical Experiments

#### 2.2.1. Sham Pain Stimulation (Nocebo Test)

The subject room contained an electronic instrument (sham stimulator), located under a computer, to create a stressful setting. Two electrodes were connected to this instrument and attached on each supraorbital region of the subject. Subjects connected to the sham stimulator were told that they would receive an undetectable electric current that increased in a stepwise fashion. For each step, patients were asked to measure the intensity of pain by pressing a number on the keyboard of the computer. Participants were also told that the current was “safe but often painful.” 

A computer program induces conditioned auditory and visual signals for the sham pain stimulus. 

Five steps, for a total time of 20 min, were expected in the experimental setting. Each step included six subsets in the computer monitor in which visual and acoustical signals were administered. 

Experimenters introduced the expectation of an increase in the intensity of the pain stimulus (sham) by (1) turning the instrument's handle, (2) increasing the acoustic intensity of the signal, and (3) saying the phrase “we are increasing the intensity of the electric current.” The signal increased at each step by 10 Hz from 110 Hz to 160 Hz. 

All subjects were instructed to use a mouse that controlled a pointer on a pain indicator displaying easily legible settings between 0 and 15. No pain corresponded to a value of 0. Pain was to be rated at 10 when aspirin or other medication for similar pain would be required (analgesic threshold). Unbearable pain corresponded to a score of 15. The total score for each step corresponds to the average of the pain score for the six subsets.

This experimental design is a modified version of the paradigm described by Bayer et al. [20, 21].

The pain intensity score for each step is the pain rating. We considered patients with nocebo response all patients who have a sum (Σ) of single step of pain rating >0.

#### 2.2.2. Cold Pressure Test

The nondominant limb was immersed in icy water (−0, 5–2°C) for a maximum of 240 sec after temperature standardization, via limb immersion, in water at body temperature (37 ± 0, 5°C) for 240 sec. We identified the time from the immersion of the limb in the icy water until the first pain sensation as the pain threshold. We defined the time from the immersion of the limb in the icy water until the limb retracted intolerable pain as the pain tolerance [22].

### 2.3. Psychological Evaluation

#### 2.3.1. SSAS

The Somatosensory Amplification Scale (SSAS) is a ten-item self-report questionnaire that measures the hypervigilance to body sensations, the tendency to select weak and infrequent sensations, and a disposition to react to somatic sensations with affect and cognitions that intensify them and make them more alarming and disturbing. Patients were asked to indicate how much each symptom had bothered them in the preceding 24 hr using a 5-point scale, with responses ranging from “not at all” to “extremely.” A higher total score indicates greater symptom amplification [23, 24].

#### 2.3.2. QUID Pain Assessment Was Conducted Using the Italian Pain Questionnaire (IPQ or QUID Questionario Italiano Del Dolore) [25]

The IPQ derives from the McGill Pain Questionnaire (MPQ); it uses the factorial structure proposed by Melzack and Torgerson [26], made up of 3 factors or classes (Sensorial, Affective, and Evaluative). 

#### 2.3.3. NRS

Pain intensity was assessed by the NRS (Numerical Rating Scale). The NRS consists of a graduate line 0–10, where the ends are labeled as the extremes of pain (no pain to excruciating pain). Patients are asked to indicate the number that best represents their intensity of pain [27].

#### 2.3.4. IBQ (Illness Behaviour Questionnaire) [28]

Scores of this questionnaire comprise 7 scales which include general hypochondriasis (phobic, anxious concern about health), disease conviction, (measures of preoccupation with symptoms and the belief that a disease is present), psychological versus somatic perception of illness (a low score suggests a tendency of somatize), affective inhibition (high scores indicate difficulty in communication of negative feeling to others), affective disturbance (presence of anxiety and depression), denial (measures the tendency to deny current life distress and to attribute all problems to physical illness), and irritability (indicates friction in interpersonal context).

#### 2.3.5. Symptom Checklist-90-Revision (SCL-90-R)

It is a multidimensional self-report symptom inventory developed by Derogatis et al. [29], and its derived Italian standard version [30] was used in this study. The SCL-90-R consists of a total of 90 questions, which are divided into nine symptom dimensions: somatization, obsessive-compulsive disorder (OCD), interpersonal sensitivity, depression, anxiety, hostility, phobic anxiety, paranoid ideation, and psychoticism. In addition to the nine dimensions, we used the Global Severity Index (GSI) representing the extent or depth of the present psychiatric disturbance. 

#### 2.3.6. MPI

The Multidimensional Pain Inventory or The West Haven-Yale Multidimensional Pain Inventory (WHYMPI) was developed in order to fill a widely recognized void in the assessment of clinical pain. Three parts of the inventory, comprised of 12 scales, examine the impact of pain on the patients' lives, the responses of others to the patients' communications of pain, and the extent to which patients participate in common daily activities. The instrument is recommended for use in conjunction with behavioral and psychophysiological assessment strategies in the evaluation of chronic pain patients in clinical settings [31]. We used the Italian version of MPI [32].

## 3. Statistical Analysis 

The data were analyzed using a StatView 5.0 software (SAS Institute Inc.). After the application of Kolmogorov-Smirnov's test that shows a Gaussian distribution of the data, we investigate the difference, in the variables, between subjects that respond to nocebo (group of patients with nocebo response) from a group of patients without nocebo response using the *t*-test analysis. We arbitrarily defined “group of patients with nocebo response” all subjects who perceived pain after an induction of false stimulation. The subject with expectation response to sham pain stimulus is defined nocebo responder when there is a sum (Σ) of average scores to 5 steps greater than 0.

## 4. Results

### 4.1. Nocebo Response

Eighty-six patients with chronic pain diagnosis were recruited from 2010 to 2011: central pain (*n* = 18), low back pain (*n* = 30), fibromyalgia (*n* = 10), headache (*n* = 16), and other diagnosis (*n* = 12) according to IASP taxonomy (1994).

**Figure 1:**
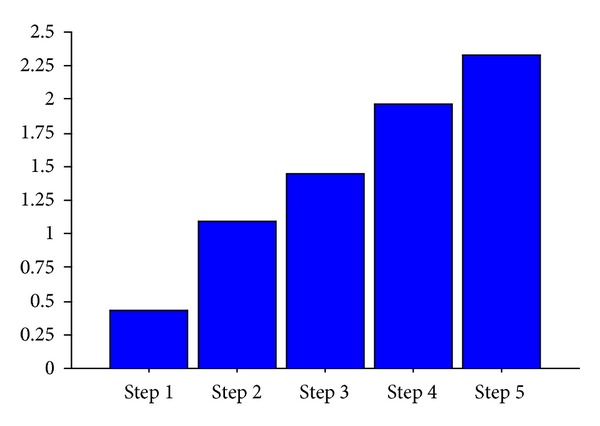
Nocebo response in chronic pain sample. ANOVA repeated measures. The mean scoring of perception of pain after a sham stimulation (nocebo response) increases to follow the steps (*F* = 16.33; df: 85; *P* < 0.0001).

**Figure 2:**
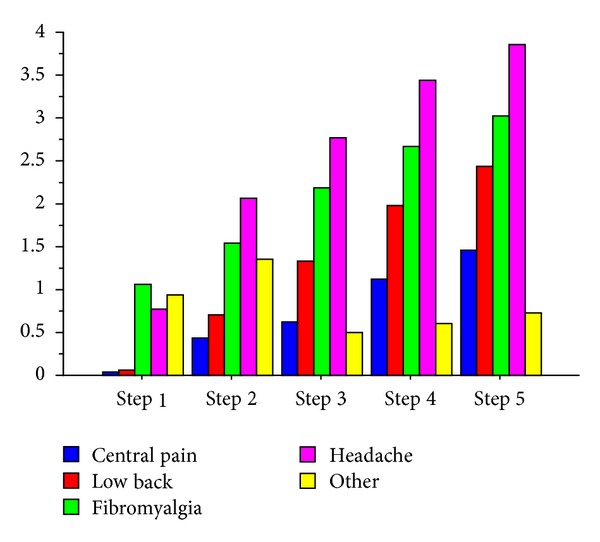
Nocebo response differences between groups of chronic pain. ANOVA between pain groups: *F* = 1.52; *P* = ns.

**Figure 3:**
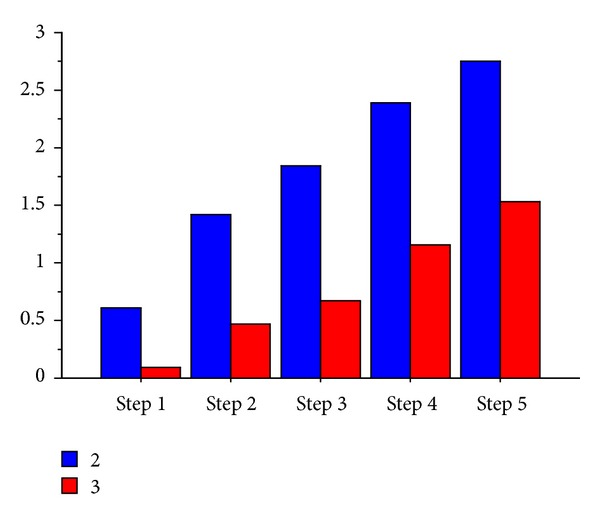
Nocebo response in the chronic pain sample separately according to sex. 2 = female; 3 = male; ANOVA between sex groups: *F* = 0.66; *P* = ns.

The nocebo response increases by going forward with the steps (*F* value 9.81; df: 85; *P* < 0.0001) as shown in Figure 1. Slight but not statistically significant differences were found between pain groups (Figure 2). Thirty-seven are patients with nocebo response (43%) and 49 (57%) are patients without nocebo response. The NRS and cold pain threshold differ between these two groups (Table 1). Female patients have slightly higher but not statistically significant nocebo response than males (Figure 3).

### 4.2. Relationship between Nocebo Response and Other Psychosocial Variables

The nocebo response depends on the presence of psychological variables, at a great nocebo response corresponds to an increase of scoring of somatosensory amplification, hypochondria, disease conviction, somatization, life control distress, support, and outdoor work (Table 2). 

### 4.3. Gender and Nocebo Response on the Management of Pain

Hypochondriasis and disease conviction of IBQ are two psychosomatic dimensions that influence the nocebo response independently of the gender. Somatization of SCL 90, life control, distress, outdoor activity, and support of MPI are dimensions that influence the increase of nocebo response in all patients but more in one of the two sexes (Table 2).

### 4.4. Nocebo Phenomenon and the Outcome of Analgesic Treatment

Only 3 patients did not continue the treatment for chronic pain up to the 6th month from baseline evaluation. In addition to the nocebo response, other psychosocial dimensions influence the outcome to analgesia. The greater the nocebo response, the higher the relief of pain (T0–T6 of NRS). The relationship between nocebo phenomenon and analgesia is linked to the presence of other psychosocial variables. As shown in Table 3, the pain relief of the total sample depends on the presence of higher intensity of pain, nocebo response, low tolerance of pain, low affective disturbance, low denial, and low somatization (Table 3). 

## 5. Discussion

We investigate the nocebo phenomenon in a clinical setting through an experimental session. Kennedy WP; where are induced an expectation of pain during a sham electrical stimulation in the eyebrows site.

In which was induced an expectation of pain during a sham electrical stimulation in the eyebrows site. This session was carried out before the beginning of a pain treatment.

We observe an increase of intensity in nocebo response during the steps administration of sham pain stimulus. Pain rating at the 5th step is higher than that at the first step (Figure 1). This data suggests an amplification of perception of stimulus with the increase of expectation (e.g, administered through the higher intensity of sound and by listening to the sentence “we are increasing the intensity of the electrical current”): when expectation increases, most pain is perceived during the sham stimulation. However, this phenomenon happens just in a part of the sample. Only 43% of chronic pain patients respond positively to the expectation of pain. Nocebo response does not differ if patients have different syndromes of chronic pain (Figure 2) or if they belong to different genders (Figure 3). The group of subjects with nocebo response (that felt pain in at least one of the sets administered) show at baseline higher pain intensity and lower cold pain threshold than group of subjects with any response to sham pain stimulus (nocebo nonresponders) (Table 1). We found no differences in the other psychosocial dimensions between these two groups.

Psychosocial dimensions affect the increase of nocebo response. In fact, some somatoform dimensions like somatic amplification, hypochondriasis, and disease conviction influence positively the strength of nocebo in those predisposed to it. Some dimensions of pain coping also affect the power of nocebo. We found a difference between sexes on the influence of all of these dimensions on the power of nocebo phenomenon (Table 2). The group with nocebo response have better but not statistically significant pain relief than a group of patients without nocebo response (NRS T0-T1, resp.: 2.79 versus 1.47; mean diff. 1.31, *t*  value = 1.81; *P* = ns).

The relevance of nocebo phenomenon has been shown in the outcome of pain treatment when associated with other psychosomatic variables. The greater is the response to the suggestion expectation of pain (we have called this “Δ nocebo”), and the higher is the effectiveness of pain therapy (Table 3). Other nocebo-associated baseline psychophysical dimensions affect pain relief: cold pain tolerance that unexpectedly affects negatively, pain intensity that affects positively, affective disturbance that influences negatively, denial affects positively, and somatization negatively affects 6 months pain relief (Table 3). No influence on the outcome of the response to analgesics was found when analyzing the power of the response nocebo alone; it was even less considering the difference in pain relief in patients with nocebo response compared to patients without nocebo response even if the first group reported better pain relief. From our results, it can be concluded that the nocebo phenomenon seems to be an “all or nothing” and this seems to be closer and stronger when there are other dimensions of the somatoform spectrum.

## 6. Conclusion

There are not many studies that correlate the nocebo phenomenon in a clinical setting. Colloca and Miller say that “translational placebo research, aimed at improving patient care, should consider the nocebo effects that can negatively influence clinical outcome in addition to placebo effects. Indeed, nocebo responses are common in clinical trials and practice and can produce discontinuation of trial participation, alteration of treatment schedules and lack of adherence” [33]. In contrast to what these two authors declare, results of our study show that the nocebo is a phenomenon which may arise or not; besides, the expectation of having pain from a false painful stimulus has not been found in all patients with pain, but only in some of them. Furthermore, in contrast to what one might expect, the presence of the nocebo phenomenon affects positively pain relief and the outcome of pain treatment. We might think that people who tend to be more alert, therefore predisposed to anxiety, may be probably the ones who have a nocebo response. But among the surveys we also evaluated the anxiety dimension (in the SCL 90) and there were no correlations. Our study shows that the nocebo phenomenon seems to be a dimension that has to be studied in a better way in psychosomatic medicine. According to our results, the new criteria proposed for the diagnosis in psychosomatic medicine (in DSM V) should include the nocebo phenomenon in the evaluation. 

Despite being known a sexual dimorphism of medically unexplained symptoms, it is still difficult to understand the psychophysiological mechanism that underlies it [34, 35]. Our results suggest a close link between psychosocial variables such as distress, somatization, social support, and the nocebo response. This link appears to differ between the sexes. This evidence suggests a trend to increase psychophysical investigation in this field that includes the impact of gender on psychosomatic dimensions and relationships between them. 

In a clinical setting, the meaning of nocebo response does not seem to be different from placebo response in the outcome of pain treatment. Both positively affect the response to the treatment. 

## Figures and Tables

**Table 1:** Differences between intensity of pain (NRS) and pain threshold in the sample distinguished by nocebo response.

	Patients with nocebo response (*n* = 37)		Patients without nocebo response (*n* = 49)		*t* value	*P*
	xM	sD	xM	sD
NRS	8.08	1.65	6.98	1.96	2.75	0.007
Cold pain threshold (sec)	**14.75**	**11.90**	**27.22**	**30.49**	**2.18**	**0.032**

NRS: numerical rating scale; Nocebo response: when a sum (∑) of mean of pain rating scoring of each of the 5 steps is > 0.

**Table 2:** Analysis of covariance (ANCOVA) using the variation of  nocebo response as a dependent variable and psychosocial dimensions as covariate.

	Δ nocebo		Δ nocebo/sex	
	*F* value/Λ	*P*	*F* value/Λ	*P*
Total SSAS	**5.62**	**0.018**		
General hypochondriasis	20.10	<0.0001		
Disease conviction	**11.51**	**0.0008**		
Somatization	9.34	0.0024	22.85	<0.0001
Life control	5.12	0.024	16.93	<0.0001
Distress	23.91	<0.0001	22.17	<0.0001
Support	**29.82**	<**0.0001**	**9.31**	**0.002**
Outdoor work	4.79	0.029	7.02	0.008

Λ: lambda; total SSAS: Somato Sensory Amplification Scale total scoring; Δ nocebo/sex: variation of  nocebo response between sexes.

**Table 3:** Effect of multiple psychosocial variables on the outcome of 6 months pain treatment.

	NRS (T0–T6)		
	*r* ^2^	*F*	*t*
Baseline independent variables (*n* = 11)	0.60	35.45****	
Nocebo (Δ)			2.11*
Cold pain threshold			1.86
Cold pain tolerance			−3.55***
Intensity of pain (NRS T0)			12.72****
SSAS total			0.79
General hypochondriasis			**0.75**
Disease conviction			1.85
Affective disturbance			−4–44****
Denial			3.22**
Irritability			−0.85
Somatization			−9.17****

Multiple regression analysis: *r*: regression coefficient; Δ nocebo: variation of nocebo response between steps; total SSAS: Somato Sensory Amplification Scale total scoring.
